# Calculating Intake of Dietary Risk Components Used in the Global Burden of Disease Studies from the What We Eat in America/National Health and Nutrition Examination Surveys

**DOI:** 10.3390/nu10101441

**Published:** 2018-10-05

**Authors:** Victor L. Fulgoni, Taylor C. Wallace, Katerina S. Stylianou, Olivier Jolliet

**Affiliations:** 1 Nutrition Impact, LLC, 9725 D Drive North, Battle Creek, MI 49014, USA; 2 Department of Nutrition and Food Studies, George Mason University, Fairfax, VA 22030, USA; twallac9@gmu.edu; 3 Think Healthy Group, Inc., 127 U Street, NW, Washington, DC 20001, USA; 4 Environmental Health Sciences, School of Public Health, University of Michigan, Ann Arbor, MI 48109, USA; kstylian@umich.edu (K.S.S.); ojolliet@umich.edu (O.J.)

**Keywords:** Global Burden of Disease, WWEIA, NHANES, sustainability, nutrition

## Abstract

Disability adjusted life years (DALYs) is a health burden metric that combines years of life lost due to disease disability and premature mortality. The Global Burden of Disease (GBD) has been using DALYs to determine the health burden associated with numerous health risks, including risks associated with dietary intakes, at the global and national level. To translate such information at the food level in the U.S., variables in What We Eat in America (WWEIA) need to be aligned with those in the GBD. In this paper, we develop the necessary new variables needed to account for differences in definitions and units between WWEIA and the GBD. We use the Food Patterns Equivalents Database, Food Patterns Equivalents Ingredient Database, Food and Nutrient Database for Dietary Studies, and Standard Reference databases that provide data for WWEIA to develop food group and nutrient variables that align with definitions and units used in the GBD. Considerable effort was needed to disaggregate mixed dishes to GBD components. We also developed a new “non-starchy” vegetable variable, since the GBD vegetables do not include potatoes and corn, and we report fruits and vegetables in grams instead of household measures. New fiber variables were created to avoid double counting of fiber from legumes, whole grains, fruits, and vegetables. Regression analyses were used to predict trans-fat content for foods in WWEIA with missing or incomplete information. The majority of foods in various U.S. Department of Agriculture (USDA) categories contain multiple GBD food groups (e.g., vegetables, whole grains, and processed meat). For most nutrients considered in the GBD, composition is more evenly distributed across the main food categories; however, seafood omega-3 fats were predominantly from either protein foods or mixed dishes and sugar sweetened beverages were from a single category. Dietary intakes in the U.S. fall short of recommendations for all food groups/nutrients with established theoretical minimum-risk targets in GBD. To our knowledge, this is the first approach that aligns WWEIA intake variables with those used in the health burden-based GBD reports. These methods will facilitate researchers to begin comparing data from the U.S. with that from other countries, as well as assess food sustainability performances by concomitantly evaluating DALYs for environmental and nutritional impacts.

## 1. Introduction

Sustainability and nutrition have become a priority interest for not only the United Nations (UN) but also many underlying states, non-governmental organizations, and the food and agricultural industries. The UN is currently promoting and coordinating implementation of internationally agreed upon development goals, including 17 Sustainable Development Goals with 169 targets within the 2030 agenda [1]. These goals and targets will stimulate action over the next decade in areas of critical importance for humanity and the planet. 

As many scientists contemplate the role of agriculture in feeding 7.2 billion people currently and 9 billion by 2040, there is a greater emphasis on food systems [2]. The food system—all the processes involved in feeding individuals across the globe—has been estimated to be responsible for approximately 25% of global greenhouse gas emissions, with significant environmental impacts on water usage, deforestation, biodiversity loss, and land degradation [3]. On top of these environmental challenges, obesity and diet-related non-communicable diseases continue to escalate, while hunger and micronutrient deficiencies continue to persist globally. There is more than enough food produced in the world to feed everyone, yet 815 million people go hungry, 155 million children are considered undernourished, and 2.1 billion people suffer from micronutrient deficiencies [4]. There is clearly a need for a more comprehensive approach to functioning food systems that are more globally sustainable and nutritious for all individuals. 

**Figure 1:**
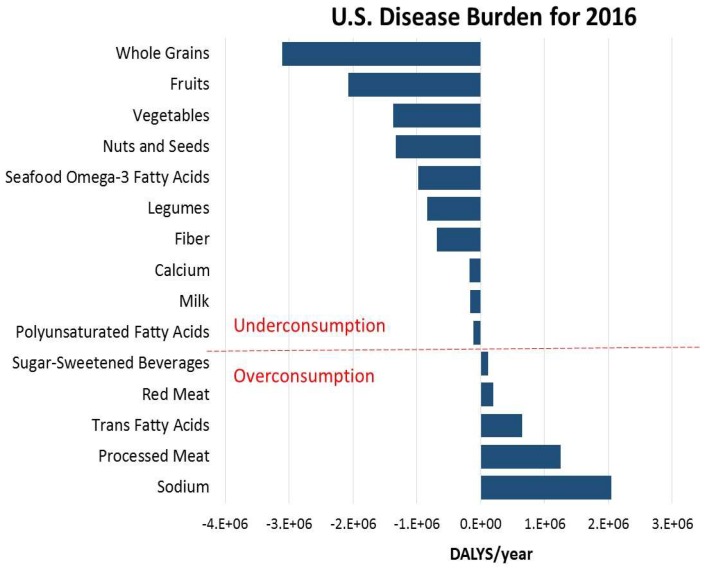
Health burden estimates for dietary risks in 2016 GBD for both sexes and all-ages as obtained from [8]. Underconsumption items indicate DALYs from not consuming the recommended amount of a component, while overconsumption indicates DALYs from exceeding recommended intakes. GBD: Global Burden of Disease; DALYs: Disability adjusted life years.

Few groups have concomitantly assessed the nutritional benefits of foods along with their environmental impact. We developed a conceptual model of how to combine nutritional and life cycle environmental impacts on human health using a common metric, disability adjusted life years (DALYs) [5]. DALYs are a time-based health burden measure that combines years of life lost due to premature mortality and years of morbidity [5]. The Global Burden of Disease (GBD) studies [6,7] have delineated DALYs for several dietary health risks that include food groups and essential nutrients (Figure 1). According to GBD estimates, in 2016 dietary risks were responsible for about 10 million DALYs in the U.S. that are associated with overconsumption of food groups and nutrients with adverse health effects and underconsumption of food groups and nutrients with beneficial health effects [8]. The dietary risk components considered by the GBD cover nine main food groups (milk, nuts and seeds, processed meat, red meat, sugar sweetened beverages, non-starchy vegetables, legumes, fruits, and whole grains) and seven nutrients (calcium, fiber, seafood omega-3 fatty acids, sodium, trans fatty acids, polyunsaturated fatty acids, and saturated fatty acids). GBD estimates are based on total diets and have not been extended to individual foods, though clearly individual foods make up the total diet and intake of dietary risk components.

While nutrient intakes from foods are fairly easily identified in dietary surveys such as What We Eat in America (WWEIA) (the dietary component of the National Health and Nutrition Examination Survey (NHANES)), food group definitions can vary by and within countries. This is a problem for determining GBD-based nutritional impacts of U.S. food items, since the WWEIA/NHANES data are currently not aligned with the dietary risk definitions used in the GBD. While conversion for products like whole fruits and vegetables is fairly simple, mixed dishes with small amounts of several ingredients that may fall in different GBD risks present significant challenges to quantifying the amounts of all of the risk components. Thus, the objective of this work was to develop methodologies to align WWEIA/NHANES food composition to GBD food groups and nutrients identified as risk factors. We aimed to determine updated measures of intakes that are consistent with the GBD risks and could be used in evaluating the health burden of the U.S. diet for individual foods/food groups. Longer term, these methodologies can be used to estimate DALYs-based sustainability indices for foods and diets that takes into account both environmental and nutritional health impacts using nationally representative data in the US.

## 2. Materials and Methods 

### 2.1. Data Sources

The National Center for Health Statistics (NCHS) of the U.S. Centers for Disease Control and Prevention (CDC) administers and collects the NHANES, a nationally representative, cross-sectional survey of noninstitutionalized civilian U.S. residents [9]. WWEIA is the dietary intake component of NHANES. Data from NHANES 2007–2008, 2009–2010, 2011–2012, and 2013–2014 were combined for development of this methodology using individuals 19+ years of age and excluding pregnant/lactating women. Questionnaires, data sets, and related documentation from each NHANES cycle can be found on the NCHS NHANES website [9]. The GBD studies, developed and implemented by the Institute for Health Metrics and Evaluation (IHME) at the University of Washington, provide a tool to quantify for 195 countries health loss from hundreds of diseases, injuries, and in particular for multiple dietary risk factors so that the global burden of disease can be reduced. The dietary risk components considered by the GBD cover nine main food groups and seven nutrients. Food groups include milk, nuts and seeds, processed meat, red meat, sugar sweetened beverages (mediated through body mass index), non-starchy vegetables, legumes, fruits, and whole grains. The nutrients considered are calcium, fiber, seafood omega-3 fatty acids, sodium (mediated through systolic blood pressure), trans fatty acids, polyunsaturated fatty acids, and indirectly saturated fatty acids (mediated through total serum cholesterol). Mediated risks listed above indicate that the health effect of the risk is associated with a diet-related metabolic risk which in turn induces adverse effects on human health. Data, analyses, methodologies, and results can be found in multiple publications (e.g., [6,7]) and on the GBD website [8]. Table 1 provides a summary description of each risk factor considered.

### 2.2. Aligning Food Groups and Nutrient Definitions with GBD Variables 

The U.S. NHANES, Food Patterns Equivalents Database (FPED) [10], Food Patterns Ingredient Database (FPID) (a component of FPED), Food Nutrient Database for Dietary Studies (FNDDS) [11], and Standard Reference (SR) [12] data were used to calculate gram weight of FPID/FPED variables for all SR foods and WWEIA food codes consumed. The FPED methodology and user guide along with FPED data were used to obtain ratios to convert FPED units of household measures such as cup, cup equivalent, ounce equivalent, etc. to grams. Methods for generation of these data can be found in the FPED Methodology and Users Guide [10]. The last column of Table 1 provides an overview of which source was used for which nutrient or food group category. In development of these methodologies, for each main FPED group variable, SR codes were categorized into groups with similar formulas for conversion. Table 2 shows the conversion group and conversion formula developed to report FPED groups in grams. Most specific foods in the SR database use a single conversion ratio to convert directly from the FPED variable to grams. Some foods use multiple conversion ratios for different parts of the food. For example, in order to calculate total vegetables (FPED group) in pizza with vegetables toppings (a WWEIA food code), we used a different conversion ratio for the pizza sauce part of the food than for each of the vegetable toppings.

Several new variables by food code and by each food code/SR combination were developed to account for differences in the WWEIA/NHANES data and that contained within the GBD database (major differences in the databases previously described in Table 1). The terms “vegetable” and “fruit” in the GBD specifically refer to the calculated variables described in Table 3. Thus, to align WWEIA vegetable determination with GBD criteria, all starchy vegetables need to be removed along with pickled and fermented vegetables. Additionally, vegetable juices are not considered as vegetables in the GBD and thus have to also be excluded from WWEIA data (including that present in vegetables packed in juice). To align WWEIA fruit determination with GBD criteria, pickled and salted fruit were excluded. Finally, the GBD evaluates separately fiber from fruits, vegetables, legumes, and whole grains and fibers from other sources since they are associated with different health outcomes. To address these differences, we developed a new variable (i.e., fiber—other sources) that determines only fiber from foods not considered as fruits, vegetables, legumes, and whole grains as defined by the GBD. In another variable, we report the fiber from fruits, vegetables, legumes, and whole grains determined as the difference between fiber as reported in WWEIA and fiber—other sources. For each component of this new fiber definition, we created a ratio of fiber for each component (i.e., refined grains, fruit not included in GBD fruit determination, and vegetables not included in GBD vegetable determination) which was applied to each SR code (Table 3).

Dietary intakes of all GBD components were estimated for adults aged 19+, 19–50, and 51+ years of age for gender combined and males/females separately. Intakes were calculated using day one intake data and adjusting for the complex sampling design of NHANES and using the corresponding sample weights. Calculations were carried out with SAS (Cary, North Carolina; version 9.4). (*p* < 0.01) across age groups and across genders within age groups were assessed using z-scores.

### 2.3. Complementing Trans-fat Data

Trans-fat data were available for about 37% of SR items, which causes 63% of food codes in WWEIA to have missing or incomplete trans-fat information. To complete a trans-fat database, we conducted linear regression analyses using SAS (version 9.4) of existing data using food group information from FPED and available nutrient information from FNDDS to create an equation to estimate trans-fat in all foods in WWEIA. Thirty U.S. Department of Agriculture (USDA) food categories out of over 150 (e.g., various fruits, various beverages, etc.) did not have any trans-fat values in the SR and 27 were assumed not to contain any trans-fat (see Supplemental Table S1). There were fourteen USDA food groups, three USDA food categories, and 12 nutrients with significant (*p* < 0.01) relationships with trans-fat in the final regression model (see Supplemental Table S2 for regression coefficients for the final model). While the final model explained over 69% of the variation in trans-fat values, when we examined predicted trans-fat values for various milk and yogurt products it appeared we were underestimating trans-fat levels, so we decided to further adjust trans-fat values for these foods by taking the ratio of known trans-fat values to total fat values for similar milk/yogurt products to determine trans-fat levels (see Supplemental Table S3 for ratios used).

## 3. Results

### 3.1. Composition of GDB Variables in USDA Main Categories of Food

Using eleven of USDA’s 15 main food categories (eliminating water, alcoholic beverages, infant formula and baby foods, and other), we profiled the foods in each category using GBD food group (Table 4) and GBD nutrients (Table 5). For some GBD food group components, a single food category was the entire source of the GBD component (i.e., beverages, non-alcoholic for sugar sweetened beverages), while other GBD components were more typically found throughout the food categories (i.e., fruits, vegetables excluding legumes, and milk, which is used as an ingredient in many other food categories) (Table 5). For most GBD nutrient components, composition was distributed across the main food categories; however, omega-3 fats were predominantly from either protein foods or mixed dishes.

### 3.2. Composition of GDB Variables in Select Foods

**Figure 2:**
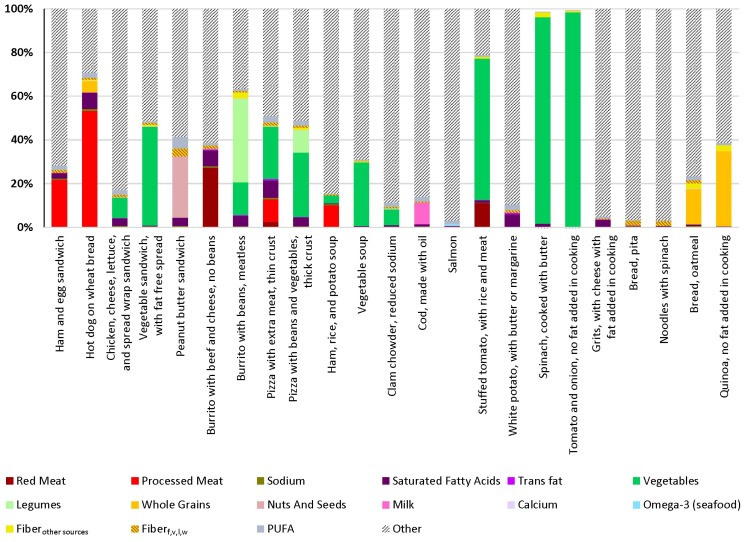
Percentage of mass of GBD dietary risk components in a serving of select foods. Data from WWEIA as modified to be consistent with GBD dietary risks. Fiber_other sources_: fiber from sources other than fruits, vegetables, legumes, and whole grains; Fiber_f,v,l,w_: fiber from fruit, vegetables, legumes, and whole grains.

GDB components in foods were summarized by quantifying percentage of total mass coming from each GBD component. Figure 2 presents this partitioning for selected items from multiple food groups. This approach allows for the delineation of the amount of GBD components and identification for which GBD components are present in predominate amounts. As expected, individual foods do not typically contain all GBD components. However, most foods contain several GBD components that can have beneficial and detrimental health effects. For example, the hot dog on wheat bread (Figure 2) contains substantial amounts of components detrimental to health such as processed meat and saturated fat but also contains some levels of some components beneficial to health in lower amounts (whole grain and polyunsaturated fat). Some of the other examples shown have meaningful amounts of specific components, for example, nuts and seeds for the peanut butter sandwich and whole grains for quinoa. This approach also provides examples of how particular foods within a food category can impact the presence and amount of GBD components, for example, meat pizzas contain high amounts of processed meat while vegetarian pizzas contain higher amounts of vegetables and sometimes legumes. While “burritos with beef and cheese, no beans” contain a substantial fraction of red meat, “burritos with beans, meatless” contains mostly vegetables and legumes.

### 3.3. GBD Food Group and Nutrient Intakes

Mean intakes of GBD dietary risks as measured in WWEIA/NHANES were calculated and compared to the GBD theoretical minimum-risk exposure levels (TMRELs), intakes that minimize the overall health risk (Table 6). The U.S. population mean intakes for GBD components beneficial to health (vegetables, milk, fruit, seafood omega-3 fats, fiber, whole grains, nuts and seeds, legumes, calcium, and polyunsaturated fatty acids (PUFA) were below their respective TMRELs (underconsumption). Mean intakes of GBD components detrimental to health (sugar-sweetened beverages, processed meat, red meat, sodium, and trans-fat) were above their respective TMRELs (over consumption). Men were more likely to consume higher amounts of many of the dietary risk factors as compared to women. Consumption of sugar-sweetened beverages, red meat, calcium, and sodium intakes were higher among adults 19–50 years (*p* < 0.01) than older counterparts. Fiber, whole grains, and PUFA were consumed in higher quantities by adults 51–99 years (*p* < 0.01) as compared to younger counterparts. In general, the U.S. adult population mean intakes failed to meet any of the GBD dietary minimum-risk exposure levels (Table 6).

## 4. Discussion

This study enabled us to align and profile WWEIA/NHANES data with the GBD dietary assessment approach. Significant work was needed to convert household measures of food groups in WWEIA, specifically from FPED, into gram amounts, which is the preferred unit in the GBD. Additionally, certain starchy vegetables (i.e., white potatoes and corn) needed to be removed from the vegetable group to be aligned with the GDB vegetable definition. To avoid double counting the health benefits of fiber, a new fiber variable was developed to exclude fiber from whole grains, fruit, legumes, and vegetables. Finally, given their importance on health in the GBD, trans-fat was imputed for all foods in the WWEIA using regression analyses.

With methodologies that align WWEIA with the work of the GBD, researchers can now develop methods to assess the overall health burden associated with foods and dietary patterns. In particular, with this work, researchers can establish DALYs for existing and recommended foods/food groups and dietary patterns to evaluate their nutritional health performance. This could help improve the development of dietary recommendations to improve the diets of Americans. In addition, such information could be potentially compared with DALYs associated with environmental impacts. Such a comparison could provide a more comprehensive assessment of individual foods/food patterns. provide insights on making dietary choices and substitutions toward more sustainable diets. Sustainable diets are those that encompass nutrition, economics, society, and the environment, each with their own measures and metrics. To be sustainable, foods and food patterns need to be nutrient-dense, affordable, culturally acceptable, and sparing of natural resources and the environment [13]. Environmental impacts of individual foods/food groups/dietary patterns have typically been measured in terms of greenhouse gas emissions, water use, etc., however, these impacts need to be weighed against nutrient density and health benefits/burden of individual foods/food groups/dietary patterns. The methods developed here will be an important step in accomplishing this task. At a very minimum, these methods can be used to generate comparative data to identify where certain foods might have the greatest overall impact, combining effects on health and the environment. 

While it is well known that Americans currently underconsume health beneficial food groups such as fruits, vegetables, whole grains, dairy, seafood, and nuts and seeds [14], our data confirm many of the food groups associated with disease in the GBD are still being underconsumed. There is a clear need to increase awareness among health professionals and consumers regarding the large portion of the U.S. population that does not meet (or in the case of other food/nutrient components such as sodium, processed meat, trans-fat, and sugar-sweetened beverages, exceed) levels of minimum health risk (e.g., TMRELs from the GBD). The 2015–2020 Dietary Guidelines for Americans encourages Americans to make substitutions, such as choosing nutrient-dense foods and beverages in place of less healthy choices, rather than increasing intake overall. Limitation of saturated fats, trans-fats, added sugars, and sodium intake has long been recommended in the U.S.; additionally, Americans also tend to consume higher amounts of refined grains [14]. The U.S. government has put key emphasis on better adherence to the U.S. Dietary Guidelines for Americans, as well as the U.S. Physical Activity Guidelines, in order to help promote health and reduce the risk of chronic disease.

Our study has several limitations. It is difficult to directly convert data collected from WWEIA/NHANES to some of the GBD variables. Various assumptions had to be made to align WWEIA/NHANES data with GBD. However, we provided considerable detail on how this was accomplished; such transparency makes it easy for others to replicate our work. Additionally, WWEIA/NHANES is a cross-sectional survey that is limited by estimates that rely on self-reported dietary data and we know underreporting in those overweight and/obese has been observed [15], so intake measures provided within need to be considered with these limitations. Finally, as the GBD are updated and further research is published on dietary factors that impact disease, changes may be needed.

## 5. Conclusions

To our knowledge, this is the first attempt to link WWEIA/NAHNES data with the GBD dietary risks. These methods will facilitate researchers to begin assessing the nutritional health performance of individual foods/food groups and compare those using DALY-based approaches. It is an important step to address multiple sustainability dimensions and to systematically analyze trade-offs between environmental impact and nutrition benefits/risks. Our current study suggests that dietary intakes in the U.S. fall short of recommendations for virtually all food groups/nutrients with established theoretical minimum-risk targets.

## Figures and Tables

**Table 1:** Description of GBD risk factors, their differences with the WWEIA/NHANES classification, and data sources used for estimating intakes. WWEIA: What We Eat in America; NHANES: National Health and Nutrition Examination Survey; FNDDS: Food Nutrient Database for Dietary Studies; SR: Standard reference; FPED: Food Patterns Equivalents Database.

Risk Factor	GBD Description	Difference with WWEIA/NHANES	Data Source
Food Groups	Intake measured in grams	Intake reported in household measures (e.g., cup, cup equivalents, etc.) in the FPED	Conversion Table 2
Sugar Sweetened Beverages	Beverages > 50 kcal/226.8 g, including carbonated beverages, sodas, energy drinks, and fruit drinks; excludes 100% fruit and vegetable juices	None	Individual Food files
Milk	Milk, including non-, low-, and full-fat milk, excluding soy milk and other plant derivatives	Soy milk and other plant derivatives considered as dairy servings	FPED [10] Table 2, Table 3
Whole Grains	Whole grains from breakfast cereals, bread, rice, pasta, biscuits, muffins, tortillas, pancakes, and other sources	None	FPED Table 2, Table 3
Nuts and Seeds	Nut and seed foods	None	FPED
Cured/Processed Meat	Meat preserved by smoking, curing, salting, or chemical preservatives	None	FPED
Red Meat	Beef, pork, lamb, and goat but excluding poultry, fish, eggs, and processed meats	None	FPED
Fruits	Fresh, frozen, cooked, canned, or dried, fruits. Excludes fruit juices and salted or pickled fruit	100% fruit juices and pickled fruits count as a fruit	FPED Table 2
Vegetables	Fresh, frozen, cooked, canned, or dried vegetables. Excludes starchy vegetables, vegetable juices, and salted or pickled vegetables	Potatoes and corn, vegetable juices, and pickles and pickled vegetables considered a vegetable	FPED Table 2, Table 3
Legumes	Fresh, frozen, cooked, canned, or dried legumes	None	Table 2
			
Nutrients			
Calcium	Calcium from all sources	None	FNDDS [11]
Fiber	Fiber from all sources, but considers fibers from fruits, vegetables, legumes, and whole grains separately from other sources	Fiber intake considers all sources	FNDDS Table 3
Omega-3 (Seafood)	Eicosapentaenoic acid and docosahexaenoic acid. Omega-3 fatty acids intake for seafood only	Omega-3 fatty acids intake considers all sources	FNDDS
Polyunsaturated Fatty Acids (PUFA)	Omega-6 fatty acids from all sources	None	FNDDS
Sodium	24 h urinary sodium measured in g per day	Sodium intake, mg per day	FNDDS
Trans-fat	Trans-fat from all sources	No direct estimates	SR [12] and regression analyses (Supplemental Tables S1–S3)

**Table 2:** List of formulas used in converting FPED household measure units to grams *.

Conversion Group	Conversion Formula
**Fruit** (cup equivalent)	
Fruit canned in water, light syrup, heavy syrup pack	100 × 0.65
Fruit nectar	100 × 0.4
Fruit juice, fruit in liquid form	Fruit, total (f_total) × 250
100% fruit after removal of sugar	100 − (added sugars (add_sugars) × 4.2)
Pineapple	Fruit, total (f_total) × 155
Raisins	Fruit, total (f_total) × 75
Dried apple	Fruit, total (f_total) × 45
Dried banana	Fruit, total (f_total) × 50
Dried berries	Fruit, total (f_total) × 75
Assorted berries/unspecified fruit	Fruit, total (f_total) × 145
Apples, baked or raw	Fruit, total (f_total) × 110
Dried figs	Fruit, total (f_total) × 75
Applesauce and banana mixture	Fruit, total (f_total) × 197.5
Cherries, peach	Fruit, total (f_total) × 155
Banana	Fruit, total (f_total) × 150
Fruit concentrate	Fruit, total (f_total) × 70
**Vegetable** (cup equivalent)	
100% vegetable after removal of sugar	100 − (Added sugars (add_sugars) × 4.2)
Vegetable in liquid form or in liquid food	Vegetables, total (v_total) × 245
Ketchup	Vegetables, total (v_total) × 120
Pimento	Vegetables, total (v_total) × 190
Tapioca	Vegetables, total (v_total) × 75
Mixed vegetables/potato/broccoli	Vegetables, total (v_total) × 155
Coleslaw	Vegetables, total (v_total) × 90
Olive	Vegetables, total (v_total) × 135
Onion rings	Vegetables, total (v_total) × 210
Vegetable in dried form	Vegetables, total (v_total) × 30
Pizza with sauce and other vegetables	(Vegetables, red or tomatoes (v_redor_tomato) × 245) + ((Vegetables, total (v_total) − Vegetables, red or tomatoes (v_redor_tomato)) × 155)
Lettuce and tomato combination	(Vegetables, red or tomatoes (v_redor_tomato) × 170) + (Vegetables, other (v_other × 110)) + ((Vegetables, total (v_total) − (Vegetables, red or tomatoes (v_redor_tomato-v_other)) × 155)
Corn	Vegetables, total (v_total) × 165
Sweet potato canned in syrup pack	Vegetables, total (v_total) × 200 − (Added sugars (add_sugars) × 4.2)
**Protein Food** (ounce equivalent)	
Protein foods using standard conversions	(Protein foods, meat, poultry, seafood, total (pf_mps_total) × 28.35) + (Protein foods, eggs (pf_eggs) × 50) + (Protein foods, nuts (pf_nutsds) × 14.175) + (Protein foods, soy (pf_soy) × 14.175)
Foods containing nut butter	(Protein foods, meat, poultry, seafood, total (pf_mps_total) × 28.35) + (Protein foods, eggs (pf_eggs) × 50) + (Protein foods, nuts (pf_nutsds) × 16) + (Protein foods, soy (pf_soy) × 14.175)
100% protein food after removal of added sugar	100 − (Added sugars (add_sugars) × 4.2)
Tofu	Protein foods, total (pf_total) × 62.5
Dried egg	(Protein foods, meat, poultry, seafood, total (pf_mps_total) × 28.35) + (protein foods, eggs (pf_eggs)/7.41 × 100) + (protein foods, nuts (pf_nutsds) × 14.175) + (Protein foods, soy (pf_soy) × 14.175)
**Dairy** (cup equivalents)	
100% dairy after removal of added sugar	100 − (Added sugars (add_sugars) × 4.2)
Dairy in fluid milk/yogurt form	((Dairy, milk (d_milk) + Dairy, yogurt(d_yogurt)) × 245) + ((Dairy, total (d_total) − Dairy, milk (d_milk) − Dairy, yogurt (d_yogurt)) × 40)
Dairy in dry, powder form	((Dairy, milk (d_milk) + Dairy, yogurt (d_yogurt) + Dairy, cheese (d_cheese)) × 25) + ((Dairy, total (d_total) − Dairy, milk (d_milk) − Dairy, yogurt (d_yogurt) − Dairy, cheese (d_cheese)) × 40)
Mozzarella	Dairy, cheese (d_cheese) × 42.525
Ricotta	Dairy, cheese (d_cheese) × 141.75
Processed cheese/blue cheese	(Dairy, cheese (d_cheese × 56.7)) + (Dairy, milk (d_milk) × 245) + (Dairy, yogurt (d_yogurt) × 245) + ((Dairy, total (d_total) − Dairy, milk (d_milk) − Dairy, yogurt (d_yogurt) − Dairy, cheese (d_cheese)) × 40)
Cottage cheese	100(Fruit, total (f_total) × 145) − (Vegetables, total (v_total) × 155)
Cheese with pimento	100 − (Vegetables, total (v_total) × 190)
**Grain** (ounce equivalents)	
Processed flour-based	Grains, total (g_total) × 16
Intact grain-based	Grains, total (g_total) × 28.35
**Legumes** (cup equivalents)	
Legumes, excluding dry	v_legumes × 175 or pf_legumes × 43.75
Legumes, dry	Vegetables, legumes (v_legumes) × 60 or protein foods, legumes (pf_legumes) × 15
**Added Sugar** (teaspoon equivalents)	
Added sugars	Added sugars (add_sugars) × 4.2
**Alcohol** (ounce equivalents)	
Ethanol	Alcoholic drinks (a_drink) × 14

* FPED abbreviations: f_total: total fruit servings in cup equivalents; add_sugars: added sugars in teaspoons; v_total: total vegetable servings in cup equivalents; v_redor_tomato: tomato and tomato product servings in cup equivalents; v_other: other vegetables in cup equivalents; pf_mps_total: total of meat, poultry, seafood, organ meat, and cured meat in ounce equivalents; pf_eggs: eggs and egg substitutes in ounce equivalents; pf_nutsds: peanuts, tree nuts, and seeds (excludes coconut) in ounce equivalents; pf_soy: soy products (excluding calcium fortified soy beverage and mature soybeans) in ounce equivalents; pf_total: total meat, poultry, organ meat, cured meat, seafood, eggs, soy, and nuts and seeds (excluding legumes) in ounce equivalents; d_milk: fluid milk, buttermilk, evaporated milk, dry milk, and calcium fortified soy beverage in cup equivalents; d_yogurt: yogurt in cup equivalents; d_cheese: cheese in cup equivalents; g_total: total whole and refined grains in ounce equivalents; v_legumes: beans and peas (legumes) computed as vegetables in cup equivalents; pf_legumes: beans and peas (legumes) computed as protein in ounce equivalents; a_drink: drink containing alcohol.

**Table 3:** Adjusting SR food codes GBD data: New variables by food code and by each food code/SR combination.

**Fruit ^1^**
General: ➢ Fruit, total (f_total) after removal of Fruit, total (f_total) from food codes with ‘pickle’ in the description and food codes described as salted (62121100).
**Vegetables ^2^**
General: ➢ Vegetables, (v_total) - Vegetables, legumes (v_legumes) ➢ Legumes (v_legumes) ➢ Vegetables, total (v_total) is removed from any food code in category 8408 (“olives, pickles, pickled vegetables”) with the exception of olives. ➢ Vegetables, total (v_total) is removed from any food code in sub group 70 (“100% Juice”).
For all other food codes, vegetable is adjusted at the SR code level: ➢ Vegetables, starchy, total (v_starchy_total) is removed from all SR codes. ➢ Vegetables, other (v_other) is removed from any SR code with a description that contains any of the words (relish, sauerkraut, pickle) and does not contain the word “olive”. ➢ Vegetables, total (v_total) is removed from SR codes in which the vegetable contribution is 100% juice (11540, 11578, 11655, 11886, 14119, 14633, 14635, 31035, 42266, 42267, 43365). ➢ 35% of v_total is removed from SR codes that are described as vegetables packed in vegetable juice (11531, 11885). FPED uses a 65/35 ratio of solid to liquid when addressing fruit packed in liquid, this ratio was applied to these vegetable SR codes.
**Omega-3 Fats ^3^**
General: ➢ Alpha linolenic acid (fa18:3(ALA)) + Eicosapentaenoic acid (fa20:5(EPA)) + Docosahexaenoic acid (fa22:6(DHA)) from: SR codes in SR food group 1500 (“Finfish and Shellfish Products”).
➢ Additional SR codes identified as seafood (36016, 36033, 43497, 80200, 83110, 90240, 90560, 93600). ➢ Mixed dish SR codes with seafood as the primary ingredient consist of clam chowder, cream of shrimp, fast food fish sandwich, and fast food tuna sandwich (6027, 6030, 6230, 6428, 6430, 27043, 6256, 6456, 21105, 20006, 21126).
**Milk ^4^**
General: ➢ Dairy, milk (d_milk) – Dairy, milk (d_milk) from SR codes described as soy (16139, 16225, 16230, 16168, 16227, 16231).
**Fiber ^5^**
General: ➢ Any SR code with fruit=0 and vegetable=0 and g_whole=0 use existing fiber amounts.
All other SR codes use the following formula: ➢ ((Grains, refined (g_refined) × Grains, refined, ratio (g_refined_ratio)) + (vegetable delta × Vegetable, ratio (vegetable_ratio)) + (fruit delta × Fruit, ratio (fruit_ratio))) / ((Grains, refined (g_refined) × Grains, refined, ratio (g_refined_ratio)) + (vegetable delta × Vegetable, ratio (vegetable_ratio)) + (fruit delta × Fruit, ratio (fruit_ratio)) + (Grains, whole (g_whole) × Grains, whole, ratio (g_whole_ratio)) + (vegetable × Vegetable, ratio (vegetable_ratio)) + (fruit × Fruit, ratio (fruit_ratio)))
**Sugar Sweetened Beverages**
General: ➢ All food codes in subgroup 72 (“Sweetened Beverages”).

^1^ After removal of pickled and salted fruits. ^2^ After addition of legumes and removal of starchy or pickled vegetables and juices. ^3^ From seafood sources. ^4^ After removal of plant-based dairy. ^5^ From sources other than fruit, vegetable, and whole grain. SR: standard reference; SR code: standard reference code. FPED abbreviations: v_total: total vegetable servings in cup equivalents; v_legumes: beans and peas (legumes) computed as vegetables in cup equivalents; v_starchy_total: total starchy vegetables (white potatoes and other starchy vegetables) in cup equivalents; v_other: other vegetables in cup equivalents; d_milk: fluid milk, buttermilk, evaporated milk, dry milk, and calcium fortified soy beverage in cup equivalents; f_total: total fruit servings in cup equivalents; g_whole: grains defined as whole grains and contain the entire grain kernel―the bran, germ, and endosperm; g_refined: refined grains that do not contain all of the components of the entire grain kernel.

**Table 4:** Main U.S. Department of Agriculture food categories of foods consumed in WWEIA/NHANES 2013–2014 by GBD food group.

GBD Food Groups (Per RACC)																				
	**Vegetables (ex. Legumes), g**				**Legumes, g**				**Milk, g**				**Fruit, g**				**Whole Grain, g**			
Main Food Category	Mean	Med	Min	Max	Mean	Med	Min	Max	Mean	Med	Min	Max	Mean	Med	Min	Max	Mean	Med	Min	Max
Milk and Dairy (*n* = 205)	0	0	0	1	0	0	0	0	90	0	0	247	1	0	0	71	0	0	0	2
Protein Foods (*n* = 971)	3	0	0	137	9	0	0	130	2	0	0	45	0	0	0	9	0	0	0	13
Mixed Dishes (*n* = 1231)	35	27	0	214	4	0	0	110	6	0	0	223	1	0	0	83	2	0	0	50
Grains (*n* = 520)	0	0	0	72	0	0	0	34	14	0	0	233	1	0	0	43	13	3	0	69
Snacks and Sweets (*n* = 662)	0	0	0	55	0	0	0	53	13	0	0	153	5	0	0	240	2	0	0	30
Fruit (*n* = 163)	0	0	0	7	0	0	0	0	0	0	0	20	100	113	0	280	0	0	0	0
Vegetables (*n* = 826)	62	82	0	241	0	0	0	35	2	0	0	50	0	0	0	109	0	0	0	1
Beverages, Nonalcoholic (*n* = 282)	0	0	0	39	0	0	0	0	32	0	0	301	41	0	0	240	0	0	0	6
Fats and Oils (*n* = 124)	0	0	0	11	0	0	0	0	2	0	0	30	0	0	0	3	0	0	0	0
Condiments and Sauces (*n* = 130)	15	0	0	124	1	0	0	24	2	0	0	110	1	0	0	16	0	0	0	0
	**Nuts and Seeds, g**				**Red Meat, g**				**Processed Meat, g**				**Sugar Sweetened Bev., g**							
	Mean	Med	Min	Max	Mean	Med	Min	Max	Mean	Med	Min	Max	Mean	Med	Min	Max				
Milk and Dairy (*n* = 205)	0	0	0	6	0	0	0	0	0	0	0	1	0	0	0	0				
Protein Foods (*n* = 971)	2	0	0	32	10	0	0	94	6	0	0	85	0	0	0	0				
Mixed Dishes (*n* = 1231)	0	0	0	32	12	0	0	139	5	0	0	100	0	0	0	0				
Grains (*n* = 520)	0	0	0	8	0	0	0	0	0	0	0	0	0	0	0	0				
Snacks and Sweets (*n* = 662)	1	0	0	21	0	0	0	0	0	0	0	0	0	0	0	0				
Fruit (*n* = 163)	0	0	0	9	0	0	0	0	0	0	0	0	0	0	0	0				
Vegetables (*n* = 826)	0	0	0	8	0	0	0	40	0	0	0	18	0	0	0	0				
Beverages, Nonalcoholic (*n* = 282)	0	0	0	3	0	0	0	0	0	0	0	0	90	0	0	360				
Fats and Oils (*n* = 124)	0	0	0	0	0	0	0	0	0	0	0	0	0	0	0	0				
Condiments and Sauces (*n* = 130)	0	0	0	10	1	0	0	23	0	0	0	8	0	0	0	0				

RACC: reference amount customarily consumed as defined by the U.S. Food and Drug Administration.

**Table 5:** Main USDA food categories of foods consumed in WWEIA/NHANES 2013–2014 by GBD nutrient.

GBD Nutrients (Per RACC)																				
	Calcium, mg				Sodium, mg				Polyunsaturated Fat, g				Trans-Fat, g				Seafood Omega-3 Fat, g			
	Mean	Med	Min	Max	Mean	Med	Min	Max	Mean	Med	Min	Max	Mean	Med	Min	Max	Mean	Med	Min	Max
Milk and Dairy (*n* = 205)	254	268	1	504	171	150	2	990	0.3	0.2	0	6	0.1	0.1	0	1.1	0.0	0	0	0.0
Protein Foods (*n* = 971)	32	17	0	348	357	329	0	2108	1.8	1.4	0	22	0.2	0.1	0	2.5	0.1	0	0	1.9
Mixed Dishes (*n* = 1231)	102	63	0	707	675	663	5	6951	2.6	2.1	0	11	0.3	0.2	0	3.9	0.0	0	0	1.2
Grains (*n* = 520)	82	35	0	1333	272	254	0	1141	1.0	0.6	0	9	0.1	0.0	0	1.5	0.0	0	0	0.0
Snacks and Sweets (*n* = 662)	52	26	0	389	149	123	0	1127	1.7	1.1	0	16	0.5	0.1	0	6.1	0.0	0	0	0.0
Fruit (*n* = 163)	19	13	0	203	15	3	0	931	0.7	0.1	0	13	0.0	0.0	0	1.3	0.0	0	0	0.0
Vegetables (*n* = 826)	44	27	2	536	254	235	1	968	0.8	0.4	0	11	0.1	0.1	0	2.0	0.0	0	0	0.1
Beverages, Nonalcoholic (*n* = 282)	83	22	0	467	64	25	0	607	0.1	0.0	0	3	0.0	0.0	0	0.8	0.0	0	0	0.0
Fats and Oils (*n* = 124)	8	3	0	44	105	81	0	480	1.9	0.5	0	11	0.3	0.1	0	2.1	0.0	0	0	0.0
Condiments and Sauces (*n* = 130)	19	7	0	189	264	173	0	3098	0.6	0.1	0	8	0.1	0.0	0	1.9	0.0	0	0	0.2

RACC: reference amount customarily consumed as defined by the U.S. Food and Drug Administration.

**Table 6:** Mean intakes of dietary risk factors in the United States as measured in WWEIA/NHANES compared to the Global Burden of Disease theoretical minimum-risk exposures.

		Age 19+ years			Age 19–50 years			Age 51+ years		
Dietary Risk Factor	GBD ^1^	All	Men	Women	All	Men	Women	All	Men	Women
SSB (g/d)	0–5	306 ± 15	376 ± 25	239 ± 14 †	411 ± 19 *	496 ± 32	321 ± 20 †	175 ± 13	209 ± 20	133 ± 12 †
Vegetables, excluding legumes (g/d)	290–430	153 ± 3	161 ± 5	146 ± 5	157 ± 3	163 ± 7	150 ± 5	149 ± 5	159 ± 8	140 ± 6
Legumes (g/d)	50–70	19.4 ± 0.9	23.7 ± 1.1	15.1 ± 0.9 †	21.4 ± 1.2	24.4 ± 1.6	18.2 ± 1.1 †	16.8 ± 1.3	22.8 ± 1.6	11.6 ± 1.6 †
Milk (g/d)	350–520	164 ± 5	195 ± 10	134 ± 3 †	166 ± 10	200 ± 18	129 ± 6 †	162 ± 7	188 ± 9	139 ± 7 †
Fruit (g/d)	200–300	152 ± 6	154 ± 7	149 ± 7	142 ± 5	141 ± 5	145 ± 8	163 ± 8	173 ± 12	155 ± 80
Omega-3 (g/d)	0.2–0.3	0.07 ± 0.01	0.08± 0.01	0.07 ± 0.01	0.07 ± 0.01	0.08 ± 0.01	0.08 ± 0.01	0.08 ± 0.01	0.08 ± 0.01	0.08 ± 0.02
Fiber (g/d)	19–28	7.8 ± 0.1	9.0 ± 0.2	6.6 ± 0.1 †	8.2 ± 0.2 *	9.3 ± 0.3	7.0 ± 0.1 †	7.3 ± 0.2	8.5 ± 0.3	6.2 ± 0.2 †
Whole Grains (g/d)	100–150	18.8 ± 0.7	20.4 ± 1.1	17.2 ± 0.5 †	16.1 ± 0.9 *	17.6 ± 1.3	14.5 ± 0.6	22.2 ± 0.9	24.4 ± 1.6	20.2 ± 0.6
Nuts and Seeds (g/d)	16–25	11.3 ± 0.8	12.5 ± 1.0	10.1 ± 0.8	9.9 ± 1.1	10.7 ± 1.4	8.7 ± 0.9	13.2 ± 1.5	15.0 ± 1.9	11.6 ± 1.8
Red Meat (g/d)	18–27	40.6 ± 1.1	51.9 ± 2.2	29.5 ± 1.2 †	46.1 ± 1.7 *	58.8 ± 2.7	32.5 ± 1.8 †	33.7 ± 1.2	42.4 ± 2.2	26.1 ± 2.0 †
Processed Meat (g/d)	0–4	28.4 ± 1.5	35.6 ± 2.5	21.4 ± 1.1 †	30.0 ± 2.0	37.8 ± 3.2	21.7 ± 1.4 †	26.5 ± 1.6	32.5 ± 2.5	21.1 ± 1.6 †
Calcium (mg/d)	1000–1500	958 ± 12	1078 ± 18	840 ± 11 †	1014 ± 17 *	1151 ± 25	868 ± 14 †	888 ± 17	977 ± 25	809 ± 19 †
PUFAs (% of energy)	9–13	8.0 ± 0.1	7.7 ± 0.1	8.2 ± 0.1 †	7.8 ± 0.1 *	7.6 ± 0.1	8.1 ± 0.1 †	8.2 ± 0.1	7.9 ± 0.2	8.5 ± 0.1
Sodium (mg/d)	1000–5000	3435 ± 32	3977 ± 52	2904 ± 23 †	3658 ± 34 *	4213 ± 58	3067 ± 31 †	3154 ± 47	3649 ± 73	2716 ± 33 †
Trans-Fat (% of energy)	0–1	0.91 ± 0.01	0.90 ± 0.01	0.92 ± 0.02	0.88 ± 0.01 *	0.87 ± 0.02	0.90 ± 0.02	0.94 ± 0.02	0.95 ± 0.02	0.94 ± 0.02

SSB: sugar-sweetened beverages; GBD: Global Burden of Disease. ^1^ Theoretical minimum-risk exposures extracted from [6]. † Means are different between men and women within each age group, *p* < 0.01 as assessed via z-scores. * Means are different across age groups, *p* < 0.01 as assessed via z-scores.

## Supplementary Materials

The following are available online at http://www.mdpi.com/2072-6643/10/10/1441/s1, Table S1: Food Categories with Zero Trans-Fat Values, Table S2: Regression Model to Estimate Trans-Fat Values for Foods with Missing Values, Table S3: Ratios of Trans-fat to Total Fat Used to Estimate Missing Trans-fat Values of Milk and Yogurt Products.
