# Supplementary material for: Calculating Intake of Dietary Risk Components Used in the Global Burden of Disease Studies from the What We Eat in America/National Health and Nutrition Examination Surveys
Source: Nutrients. 2018 Oct 5;10(10):1441. doi: 10.3390/nu10101441 (PMC6212931; doi:10.3390/nu10101441)
Supplement: Supplementary file 1 [file nutrients-10-01441-s001.pdf]

Supplemental Table 1. USDA food categories with zero trans-fat values.

Category Number	Category Description
6012	Citrus fruits
6014	Melons
7004	Apple juice
7102	Diet soft drinks
7104	Diet sport and energy drinks
7106	Other diet drinks
7202	Soft drinks
7206	Sport and energy drinks
7502	Beer
7504	Wine
7702	Tap water
7704	Bottled water
7802	Flavored or carbonated water
7804	Enhanced or fortified water
8404	Soy-based condiments
8802	Sugars and honey
8804	Sugar substitutes
9004	Baby food: fruit
9006	Baby food: vegetable
9010	Baby food: yogurt
9202	Baby juice
9204	Baby water
9402	Formula, ready-to-feed
9404	Formula, prepared from powder
9406	Formula, prepared from concentrate
9602	Human milk
9802	Protein and nutritional powders

Supplemental Table 2. Regression model to estimate trans-fat values for foods with missing values.

Variable	Beta Coefficient	Standard Error	P-value
2202 Chicken, whole pieces	0.2514	0.0615	<0.0001
2604 Bacon	-0.6738	0.1673	<0.0001
2804 Nuts and seeds	-0.5838	0.1171	<0.0001
3703 Frankfurter sandwiches	-0.2389	0.0724	0.0010
4208 Tortillas	-0.8442	0.1970	<0.0001
5002 Potato chips	0.4621	0.1584	0.0036
5202 Crackers, excludes saltines	-0.2918	0.0891	0.0011
5402 Cereal bars	-0.3068	0.0806	0.0001
5504 Cookies and brownies	-0.4308	0.0693	<0.0001
5506 Doughnuts, sweet rolls, pastries	2.4832	0.1214	<0.0001
5702 Candy containing chocolate	-1.0500	0.0877	<0.0001
5704 Candy not containing chocolate	-0.4582	0.1043	<0.0001
8004 Margarine	4.2649	0.1473	<0.0001
8008 Cream and cream substitutes	1.0989	0.1689	<0.0001
Oils (g)	-0.0284	0.0050	<0.0001
Seafood high in n-3 fatty acids (oz eq)	0.2964	0.0405	<0.0001

Solid fats (g)	0.0376	0.0046	<0.0001
MFA 16:1 (Hexadecenoic) (gm)	-0.9008	0.0927	<0.0001
MFA 18:1 (Octadecenoic) (gm)	-0.1001	0.0127	<0.0001
MFA 20:1 (Eicosenoic) (gm)	-2.0234	0.1902	<0.0001
PFA 18:2 (Octadecadienoic) (gm)	0.2557	0.0618	<0.0001
PFA 18:3 (Octadecatrienoic) (gm)	0.2278	0.0620	0.0002
SFA 10:0 (Decanoic) (gm)	-0.8140	0.1015	<0.0001
SFA 12:0 (Dodecanoic) (gm)	-0.2546	0.0178	<0.0001
SFA 16:0 (Hexadecanoic) (gm)	-0.3842	0.0234	<0.0001
Magnesium (mg)	-0.0006	0.0002	0.0025
Total polyunsaturated fatty acids (gm)	-0.3969	0.0622	<0.0001
Total fat (gm)	0.1799	0.0118	<0.0001
Vitamin E as alpha-tocopherol (mg)	-0.0119	0.0026	<0.0001

Stepwise regression was utilized including all USDA What We Eat In America (WWEIA) food categories, all Food Patterns Equivalents Database (FPED) food groups, and all nutrients provided in the Food and Nutrient database for Dietary Studies (FNDDS). Only those variables with $p < 0.01$ were retained.

Supplemental Table 3. Ratios of trans-fat to total fat used to estimate missing trans-fat values of milk and yogurt products.

USDA Category Number	USDA Category Description	Trans Fat to Total Fat Ratio
1002	Milk, whole	0.04302
1004	Milk, reduced fat	0.04302
1006	Milk, low-fat	0.03658
1008	Milk, nonfat	0.03658
1202	Flavored milk, whole	0.03511
1204	Flavored milk, reduced fat	0.03511
1206	Flavored milk, low-fat	0.03460
1820	Yogurt, regular	0.00923
1822	Yogurt, Greek	0.00923
